# Gut Barrier Dysfunction and Microbiota Variations in Cryptosporidiosis: A Comprehensive Review

**DOI:** 10.3390/vetsci12020085

**Published:** 2025-01-23

**Authors:** Munwar Ali, Chang Xu, Mingyue Wang, Qazal Hina, Yaru Ji, Subiha Anwar, Sijia Lu, Qing He, Yawei Qiu, Kun Li

**Affiliations:** 1 College of Veterinary Medicine, Nanjing Agricultural University, Nanjing 210095, China; drmunwarali06@gmail.com (M.A.);; 2 MOE Joint International Research Laboratory of Animal Health and Food Safety, College of Veterinary Medicine, Nanjing Agricultural University, Nanjing 210095, China; 3 Department of Animal Nutrition, University of Veterinary and Animal Sciences, Lahore 54000, Pakistan; 4 Department of Animal Husbandry, University of Agriculture, Faisalabad 38000, Pakistan

**Keywords:** *Cryptosporidium*, protozoan, zoonotic, gut, intestinal barrier, microbiota

## Abstract

Cryptosporidiosis is a zoonotic protozoan parasite-born disease, equally significant in both animals and humans, especially affecting immunocompromised individuals (e.g., AIDS patients) and neonates. The prime concerns of this review article are to demonstrate the disruption of the intestinal barrier and variations in the gut microbiome during cryptosporidiosis, and to explore host gut–parasite interactions that can lead to the development of novel therapeutics. The review concluded that the enteric barrier is particularly maintained by tight junction proteins (e.g., occludin, claudin, and ZO-1, etc.) and mucosal immunity, both of which are severely compromised during *Cryptosporidium* spp. infections, resulting in increased intestinal barrier permeability, inflammatory responses, diarrhea, and ultimately death in severe cases. *Cryptosporidium*-induced dysbiosis is characterized by reduced microbial diversity and richness, a shift from commensal to pathogenic bacteria, as evidenced by increased pro-inflammatory taxa like Proteobacteria, and reduced proportions of beneficial SCFAs producing bacteria, e.g., Firmicutes. Recent investigations have highlighted the interrelations between gut microbiota and epithelial barrier integrity, especially during cryptosporidiosis, demonstrating the modulations regarding tight junctions (TJs), immune reactions, and SCFA production, all of which are main players in alleviating this protozoal parasitic infection. This review comprehensively describes the fine details underlying these impairments, including autophagy-mediated TJs’ degradation, inflammasome activation, and gut microbiome-driven alterations in metabolic pathways, providing the latest relevant, and well-organized piece of knowledge regarding intestinal barrier alterations and microbial shifts during cryptosporidiosis. This work emphasizes the future need for longitudinal studies and advanced sequencing techniques to understand host gut microbiota–parasite interactions, aiming to formulate innovative strategies to mitigate cryptosporidiosis.

## Simple Summary

*Cryptosporidium*, a genus of zoonotic protozoans, poses a threat to one’s health through the disruption of gut microbiota and intestinal barrier functions. This review addresses the underlying mechanisms through which *Cryptosporidium* spp. impairs the gut barrier, underscoring the importance of junctional proteins (JPs), the mucosal immune response, and host microbiome–parasite interrelations. It also demonstrates how this parasitic infection causes gut microbiota alterations, focusing on the role of short-chain fatty acids (SCFAs) in alleviating infection severity. Therapeutic approaches, e.g., probiotics usage, dietary manipulations, and microbiota transplantation/restoration, are briefly discussed here, necessitating further research using advanced approaches (metagenomics or metabolomics) to control the ever-increasing global burden of cryptosporidiosis.

## 1. Introduction

*Cryptosporidium*, a genus of protozoan parasites, was first identified in the early 20th century [1], and later attracted significant attention due to its increasing prevalence, wide host range, zoonotic potential, and unique characteristics [2]. *Cryptosporidium* spp. are parasitic organisms, residing intracellularly but separated from the host cell cytoplasm due to their presence in a structure known as the parasitophorous vacuole, infecting the enteric epithelial cells in different vertebrates and resulting in cryptosporidiosis [3,4]. Cryptosporidiosis primarily infects children, newborn animals, and people with compromised immune responses. In low-income regions, cryptosporidiosis is the second leading cause of neonatal diarrhea, resulting in increased proportions of mortality in children under the age of two [5,6]. The interrelations between enterotoxic activity and diarrhea during intestinal cryptosporidiosis manifest through the secretion of an enterotoxic moiety synthesized by *Cryptosporidium* spp., which leads to lethal diarrhea, as evidenced by the enhanced short-circuit current (Isc) in Caco-2 cells exposed to fecal supernatant from infected individuals, demonstrating significant electrolyte secretion, especially of chloride [7]. Hence, cryptosporidiosis significantly contributes to food and waterborne illnesses, even in the developed world [8,9]. Out of more than 20 *Cryptosporidium* spp. and genotypes, *C. hominis* and *C. parvum* account for more than 90% of cases worldwide [10,11].

*Cryptosporidium* mostly affects cattle, water buffalo, camels, horses, sheep, goats, poultry, rabbits, pigs, donkeys, deer, wild mammals, and fish [12,13,14,15,16]. The prevalence of *Cryptosporidium* spp. infection was found to be 28.52% in cattle, 18% in buffalo calves, between 27.8 and 60.4% in pigs, 52.7% in dogs, and 29.4% in cats [17,18]. Recently, *C. parvum*’s presence in yaks has been reported in China, which may prove to be a serious food-safety threat, ultimately affecting public health [19,20,21]. Before weaning, *C. parvum* is the most common intestinal pathogen in calves [22]. One infected calf can shed up to 1 × 10^8^ oocysts in feces, posing a risk to other susceptible hosts [23]. Losses due to this disease in the cattle industry include calf mortality, diagnosis expenses, medication and supportive care, and increased market age [24]. The global load of cryptosporidiosis in animal dung is 3.2 × 10^23^ oocysts per year, to which cattle is a major contributor [25,26]. Additionally, *C. parvum* is the leading cause of diarrhea in calves and lambs [27]. Hence, *Cryptosporidium* spp. have a significant zoonotic potential in human beings due to their common interactions with livestock species [28,29,30]. Two investigations in China indicated that *C. andersoni* showed the highest prevalent *Cryptosporidium* spp. found in humans [31]. Similarly, *C. bovis* was initially detected in a 3-year-old toddler and a 23-year-old adult from two different farms in Australia in 2012, who consumed raw milk and had intensive interaction with dairy animals [32]. Another study investigated a combined *C. bovis* and *C. parvum* infection in a diarrheic young child aged less than six years from Egypt, who had contact with cattle, resulting in mixed infections [33,34]. Therefore, the potential of cattle to transmit several species of *Cryptosporidium* spp., particularly *C. hominis*, represents a major public health hazard associated with possible interactions. Its zoonotic potential is significant worldwide in both the public health and livestock sectors, especially regarding *C. parvum* IIa subtypes, which are highly prevalent in dairy calves [3,35].

To prevent this protozoan parasite, there is currently no vaccine. Nitazoxanide (NTZ) is the only drug approved by the US Food and Drug Administration (FDA) for treating *Cryptosporidium* spp. infections [36,37,38]; however, its effectiveness is limited in immunocompromised and malnourished individuals [39,40,41,42]. Paromomycin is another drug that also shows inconsistent therapeutic efficacy in people with compromised immunity [41]. Animals such as goat kids, calves [43,44], neonatal mice in their early life stage [45], and goats are highly vulnerable to *Cryptosporidium* spp. infection, while in the murine model, it was observed that vulnerability to this protozoan parasite gradually decreases with age [46]. However, the underlying mechanism for lethal diarrhea induced by *Cryptosporidium* spp. infection still lacks clarity.

The enteric defense mechanism plays an integral role in preserving gut homeostasis and protecting it from notorious pathogens, as evidenced during cryptosporidiosis [47,48,49,50,51]. *Cryptosporidium* spp. disrupt the integrity of the enteric epithelial barrier by modulating the expression of integral adherens junctions (AJ) and tight junction proteins (TJPs), which result in an enhanced enteric barrier permeability [50]. Contemporary studies have shown that *C. parvum* infection significantly decreases the key JPs, e.g., E-cadherin and occludin, which are vital to inhibit the systemic invasion of toxins and infectious agents [50,51]. The resulting gut barrier dysfunction not only manifests as diarrhea during cryptosporidiosis, but also impairs immune reactions and overall host health, particularly in highly susceptible populations [2,51]. The exploration of these mechanisms is of great significance to develop potential therapeutics for the restoration of the gut barrier function and to mitigate serious health outcomes post infection.

*Cryptosporidium* spp. infections affect the gut microbe’s composition, which plays a crucial role in host survival [52,53]. It can also lead to gut dysbiosis, characterized by reduced diversity or an increase in inflammatory or potentially pathogenic bacteria [54,55]. The host may experience long-term effects, including irritable bowel syndrome [53,56]. In addition, reduced bacterial diversity in the host gut is associated with a weakened immune response and the dominance of potentially harmful bacteria [55,57]. The immune system interacts with commensal gut bacteria through nucleotide-binding oligomerization domain (NOD)-like receptors, toll-like receptors (TLRs), and pattern recognition receptors (PRRs) [58], underscoring the role of commensal bacteria in the pathogenesis of diseases [58]. Gut microbiota can influence immune responses, potentially exacerbating or misdirecting them against pathogens like *C. parvum*, thereby impacting disease severity [58].

This review aims to investigate the dysfunction of different intestinal barrier proteins during *Cryptosporidium* spp. infection to understand their integral role during cryptosporidiosis. Also, the literature regarding gut microbiota variations during cryptosporidiosis is briefly discussed here. These discussions will provide a guide for future researchers.

## 2. Methodology

The information presented here was obtained by searching electronic internet databases like SCOPUS, Web of Science, PubMed, and Science Direct, as well as by searching Google Scholar and by visiting specific relevant journals. Various combinations of keywords, such as the *Cryptosporidium*, tight junction proteins, gut microbiota variations/perturbations, *Cryptosporidium* and gut barrier integrity, zoonotic transmission, prevalence, and future perspectives, were used. This review mainly included peer-reviewed scientific articles from world-renowned journals written in English, focusing on the most recent and relevant data throughout the manuscript. Initially, 200 articles were downloaded, and later 34 articles were excluded due to their low impact factors, their being old, and to avoid repetition.

## 3. Protective Role of the Intestinal Barrier and Gut Dysfunctioning in Cryptosporidiosis

### 3.1. Key Components of the Intestinal Barrier and Their Roles

The enteric epithelium consists of a single layer of cells forming the gut lining, serving an essential function in nutrient absorption, maintaining barrier integrity, and initiating immune reactions. It mainly comprises enteric epithelial cells, e.g., enterocytes, enteroendocrine cells, paneth cells, and goblet cells [47,48,49]. Enterocytes are dominant cells in the intestine and are integral for nutrient absorption. Their increased surface area due to the presence of microvilli on their apical surfaces (brush border) is significant in nutrient absorption and the secretion of enzymes needed for digestion. Also, the presence of goblet cells results in mucus secretion to lubricate and protect the gut mucosal lining [59]. At the base of crypts, the presence of paneth cells ensures the secretion of antimicrobial peptides, which are important players in gut immunity [60]. Enteroendocrine cells are responsible for hormone production and are involved in different physiological processes. It has been observed that there is continuous renewal of the enteric epithelium every 3 to 5 days, linking this process to stem cells located in the crypts of Lieberkühn, guaranteeing the prompt replenishment and repair of intestinal barrier integrity [60,61].

Tight junctions are a special type of intercellular junction positioned at the apical surface of intestinal epithelial cells. They are especially involved in the maintenance of the gut barrier by checking paracellular trafficking. Tight junctions consist of several crucial membrane proteins that interact to form a selective barrier. For instance, claudins are a family of proteins that form the backbone of TJ strands. Different claudins have varying permeability properties, influencing the passage of ions and small molecules. Similarly, occludin is an integral membrane protein that plays a crucial role in the structure and function of TJs, enhancing barrier integrity [62,63,64]. Junctional adhesion molecules (JAMs) facilitate cell–cell adhesion and contribute to the stability of TJs. Tricellulin, located at tricellular junctions where three epithelial cells meet, is essential for maintaining barrier integrity in these complex areas. Additionally, zonula occluden (ZO) proteins, such as ZO-1 and ZO-2, are transmembrane proteins that link TJs to the actin cytoskeleton and various signaling pathways, further supporting the structural and functional integrity of TJs [62,63,64]. This connection is vital for maintaining cell shape and regulating TJs’ dynamics.

Tight junctions prevent the passage of pathogens, toxins, and large molecules from the intestinal lumen into the bloodstream, thus maintaining gut homeostasis [64,65]. They regulate paracellular transport by controlling ions, hence allowing the passage of small molecules. This selectivity is crucial for nutrient absorption while preventing harmful substances from entering systemic circulation [62,63]. They are involved in signaling pathways that influence cell proliferation, differentiation, and immune responses [66]. However, the integrity of TJs can be influenced by various factors, including dietary components, inflammatory cytokines (e.g., IL-1β), and microbial interactions. The disruption of TJs’ integrity has been linked to various gastrointestinal diseases, such as inflammatory bowel disease (IBD), celiac disease, and cryptosporidiosis [60,63,65].

### 3.2. Enteric Histopathophysiology in Cryptosporidiosis

**Figure 1:**
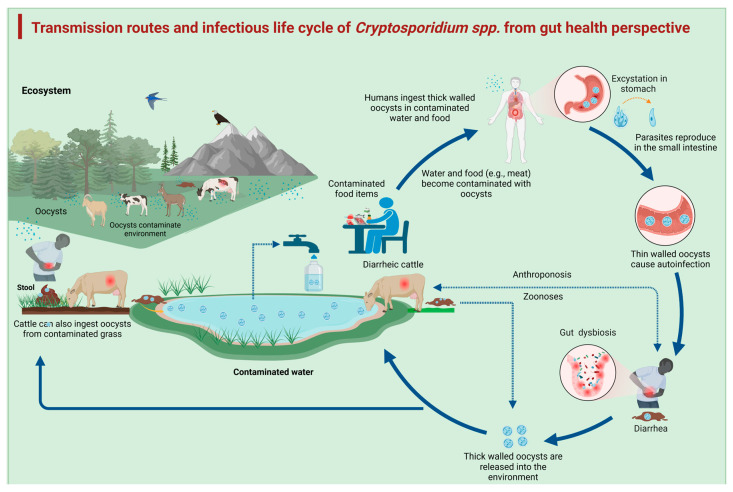
Transmission route of *Cryptosporidium* spp. from a gut health perspective. The cycle begins with the ingestion of oocysts from contaminated food and water, which leads to excystation in the stomach. The parasite reproduces in the small intestine, where thin-walled oocysts cause autoinfection, resulting in diarrhea, while the thick-walled oocysts are released into the environment and infect other susceptible hosts.

*Cryptosporidium parvum* infection causes villus atrophy, with infected tissues showing significantly shorter villi than the control group. Histological studies have showed a significant reduction in intestinal villi length, with up to 50% shortening observed during peak infection in *Cryptosporidium*-infected animals [50,51,67]. Villi blunting results from parasites attaching to enterocytes, causing dysfunction and eventual apoptosis [51]. Unlike villus atrophy, *C. parvum* infection triggers crypts’ hyperplasia, with increased crypt depth as a compensatory response to the shortening of villi. This is accompanied by enhanced crypt cell proliferation observed in infected tissues [67,68]. The hyperplastic mechanism’s objective is to regain intestinal absorptive capacity; however, it often fails to fully compensate for the enteric villi architecture loss (Figure 1).

**Figure 2:**
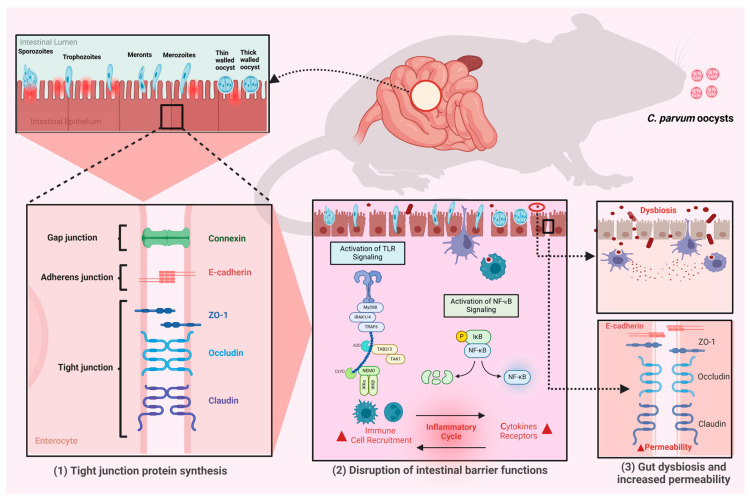
Invasion of *C. parvum* into enterocytes triggers an inflammatory response, leading to gut dysbiosis and diarrhea. The *Cryptosporidium* spp. infections disrupt the intestinal barrier by downregulating key TJPs, including ZO-1, E-cadherin, occludin, and claudin. As a result of this protozoan parasitic infection, immune cell infiltration, the activation of inflammatory cascades (TLR signaling and NF-κB pathways), and the disruption of intestinal barrier functions occur.

The villus-to-crypt ratio (V:C) is a key indicator of intestinal functionality and health in *Cryptosporidium* spp. infection; this ratio is greatly altered due to decreased villus height and enhanced crypt depth. Previous studies have demonstrated that infected animals showed a significantly lower V:C ratio than healthy controls, demonstrating compromised intestinal functioning and architecture [51,69], impairing nutrient absorption, and resulting in enhanced intestinal permeability. The process through which *Cryptosporidium* spp. negatively influence enteric morphology involves epithelial barrier impairment. The parasite binds to the apical surface of intestinal cells, leading to cellular stress that enhances epithelial permeability and promotes enteritis [70,71]. This disruption involves the downregulation of TJPs like E-cadherin, essential for the integrity of enteric epithelium (Figure 2) [51]. Damaged TJs allow pathogens and toxins to cross the epithelial barrier, worsening tissue damage followed by inflammatory cascades.

*Cryptosporidium* spp. induce strong inflammatory reactions, marked by immune cell infiltrations into the enteric mucosa. Neutrophils and released cytokines like IL-8 contribute to tissue harm and impaired epithelial regeneration [68,69]. The inflammatory mechanism promotes crypt cells’ increment while inducing apoptosis in nearby epithelial cells, playing an integral role in villi blunting [50].

### 3.3. Cryptosporidium and Gut Barrier Dysfunction

*Cryptosporidium parvum* infection significantly downregulates primary TJPs, e.g., E-cadherin, claudin-4, and occludins (Figure 2). Previous investigations using monolayers of Caco-2 cells have shown that *C. parvum* increased intercellular permeability because of the downregulation of TJPs, thereby impairing gut barrier functions. Particularly, claudin-4 and occludin were found to be significantly reduced, and other proteins (junctional), e.g., ZO-1 and claudin-3, also exhibited reduced levels [72]. Tight junctions’ disruption resulted in increased intestinal epithelium permeability, allowing pathogens and toxins to pass into underlying tissues. This was evidenced by an increased flux of fluorescein isothiocyanate (FITC)-dextran across infected Caco-2 cell monolayers, and decreased transepithelial electrical resistance (TEER) measurements [51,73].

Recent investigations have found that *C. parvum* infection positively influences autophagy in enteric epithelial cells, playing a role in the degradation of TJPs. The initiation of autophagy was associated with the reduced phosphorylation of the mammalian target of rapamycin (mTOR), clarifying the underlying mechanism where autophagy reduces the integrity of TJs via facilitating the degradation of claudin-4 and occludin [51,74]. The silencing of ATG7 (a main player in autophagy) resulted in the upregulation of TJPs, pointing out the role of autophagy in regulating this process. Additionally, the established results suggest the potential role of post-translational modifications in mediating the effects of *C. parvum*. For example, inhibitors such as bafilomycin-A have been shown to partially alleviate the cytopathic effects of *C. parvum* on occludin expression, suggesting that this protozoan parasite may trigger pathways responsible for protein degradation, resulting in decreased levels of TJ proteins [51,73].

*Cryptosporidium parvum* affects TJPs and AJPs, especially E-cadherin, which is integral for intercellular adhesion. During infection, the suppression of E-cadherin aggravates intestinal barrier dysfunction and results in the enhanced permeability of the intestine [51,75], which results in diarrhea during cryptosporidiosis. Additionally, after the initiation of inflammatory cascades, the parasite goes deeper into the tissues, further complicating the pathological cascades. Due to the impairment of intestinal integrity, nutrient absorption is negatively affected, resulting in malnutrition in affected individuals [51,76].

### 3.4. Inflammatory Cascades: Immunological Reactions Against Cryptosporidiosis

#### 3.4.1. Role of Caspase-1 in Cryptosporidiosis

Caspase-1, a main element of the inflammasome, plays a vital role in the host’s defense against *Cryptosporidium* spp. infections. In an experimental model where caspase-1 was knocked out in intestinal epithelial cells, an 18-fold parasitic burden was observed compared to wild-type mice. These findings emphasize that epithelial cell intrinsic signaling via caspase-1 is crucial for early control of *C. parvum* replication [77,78]. In particular, intrinsic activation of caspase-1 in epithelial cells has been shown to positively influence interleukin-18 (IL-18) secretion, which is important for activating immune reactions during cryptosporidiosis [79]. Studies have shown that caspase-1 modulates the expression of the gene in a tissue-dependent fashion within the enteric environment. Especially, it has been demonstrated that caspase-1 can downregulate or upregulate a variety of genes, with special reference to the type of intestinal tissue, e.g., ileum, jejunum, and duodenum. For example, a study examining gene expression in various sections of the intestine revealed that caspase-1 influences 313 common genes’ expression across the mentioned tissues [79]. This underscores the intricate role of caspase-1 in regulating inflammatory cascades during *Cryptosporidium* spp. infection.

Upon activation, caspase-1 breaks pro-IL-18 and pro-IL-1β into their active forms, which play a critical role in triggering inflammatory cascades. The release of these cytokines is crucial for coordinating the immune response to cryptosporidiosis infection [79,80]. Hence, elevated levels of caspase-1 are associated with increased resistance to cryptosporidiosis [77,81]. Additionally, the external administration of IL-18 restored parasite regulation in caspase-1-deficient mice, further clarifying its role in this context [77]. However, current studies indicate that type I interferon (IFN) signaling may impair the enteric defense against *C. parvum*. Enteric epithelial cells infected with *C. parvum* have shown alterations in gene expression patterns linked to type-I IFNs reactions, potentially compromising effective immune defenses against the parasite [81]. Although caspase-1 is essential for triggering protective responses, other signaling mechanisms may complicate host immune responses.

#### 3.4.2. Interactions Between Capsae-1 and NLRP6 in Cryptosporidiosis

Caspase-1 is crucial for the immune response to *C. parvum*, especially through its interaction with the NOD-like receptor family pyrin domain containing 6 (NLRP6). This interaction is vital for managing intestinal inflammation and enabling the host to control parasitic infections. Current studies have demonstrated the underlying mechanisms through which NLRP6 and caspase-1 aid the immune defense against *C. parvum* [77]. As an integral receptor, NLRP6 is essential for caspase-1 activation against *C. parvum*. Studies have shown that NLRP6 is essential for recognizing this parasite by the innate immune response, and that its lack results in enhanced vulnerability to infection. Mice lacking NLRP6 not only have elevated parasite loads, but also demonstrated impaired IL-18 production, which underscores the importance of this pathway in orchestrating an effective immune response(Table 1) [82,83,84]. The epithelial cells are primarily responsible for generating IL-18 in response to *C. parvum*, highlighting their role as key players in controlling infection [70]. Further studies regarding cellular processes showed that the activation of caspase-1 is especially needed within enteric epithelial cells.

The interrelation between NLRP6 and caspase-1 also impacts various other immune system elements. For example, research suggests that ASC (apoptosis-associated speck-like protein) is a key element of the inflammasome complex and is crucial in influencing susceptibility to cryptosporidiosis. Mice deficient in ASC showed an exaggerated parasite burden during acute phases of infection, but ultimately succeeded in controlling the cryptosporidiosis, indicating the possible involvement of a compensatory process, which needs to be further explored (Table 1) [78,83].

## 4. Gut Microbiota Alterations in Cryptosporidiosis: Impact and Implications

*Cryptosporidium* spp. infection in ruminants, humans, and non-human primates has been linked to reduced gut microbiome alpha diversity (Shannon Index). However, certain bacterial genera are known to increase during infection, while little is known about those that are depleted. During infection, key taxa that increased include Firmicutes [100], Pseudomonadota (formerly known as Proteobacteria) [101], different types of protozoa, and Actinomycetota [100]. In the gut ecosystem, Firmicutes are the key players in butyrate production. This phylum comprises Gram-positive bacteria such as *Enterococcus*, *Lactobacillus*, Clostridiales, and Lachnospiraceae. Increased butyrate production is linked to healthier, more diverse bacterial communities and a lower hydrogen sulfide concentration [102,103]. Firmicutes significantly increase after birth [104,105], as observed in a study of 20 neonatal calves [105]. This phylum is also considered a cornerstone of a healthy gut microbiome [106]. Another study found an increased abundance of bacteria such as *Lactobacillus* and members of Coriobacteriaceae in cryptosporidiosis, particularly in the small intestine. Coriobacteriaceae have been exhibited to regulate glucose metabolism [107]. Similarly, an increased level of *Lactobacillus* may demonstrate a gut microbiota response to compensate for the *Cryptosporidium* spp.-induced intestinal damage [100].

The Firmicutes/Bacteroidetes ratio is associated with gut flora homeostasis and serves as an indicator of an individual’s health [108]. An increased Firmicutes/Bacteroidetes ratio has been linked to obesity in both murine and human models [108,109], while it has not been linked to cryptosporidiosis severity, presenting a potential area for exploration. Alternatively, gut microbiota-associated metabolites such as fecal indole concentrations, as suggested by Chappell et al. (2016), could serve as biomarkers for susceptibility to this protozoal disease [110]. In this study, patients with higher fecal indole levels resisted *C. parvum* infection, while those with lower levels were susceptible [110]. Bacteroidota reduces fecal indole levels, indicating that the Firmicutes/Bacteroidetes ratio combined with fecal indole concentration could serve as a reliable biomarker for susceptibility to cryptosporidiosis. During cryptosporidiosis, healthy bacteria like Firmicutes decrease, while pro-inflammatory bacteria such as Proteobacteria often colonize the gut [111,112].

Intestinal infections like *C. parvum* cause gastroenteritis, which is worsened by pro-inflammatory bacteria colonization. High levels of Proteobacteria are found to be linked to inflammation, and metabolic disorders in bovine calves serve as markers of gut dysbiosis [67]. An increased Proteobacteria abundance during *C. parvum* indicates reduced gut bacterial diversity. Studies have shown that dietary modifications and probiotics can help reduce the severity of cryptosporidiosis [113,114,115]. After probiotic supplementation, *Lactobacillus* reduced oocyst output, whereas a mix of *Bifidobacter*, *Streptococcus*, and *Lactobacillus* significantly increased the oocyst output of [114,116], suggesting that outcomes depend upon host type and microbiome composition, creating further research gaps regarding host–microbiome interactions during cryptosporidiosis. Studies have shown that mice on high-fiber diets shed fewer oocysts than those on low-fiber diets, due to changes in microbiota after specific feed types, further clarifying the role of gut microbiota variations due to cryptosporidiosis [115]. Probiotics or high-fiber diets may restore the gut bacteria lost during infection, preventing pro-inflammatory bacterial colonization and alleviating cryptosporidiosis symptoms. These interventions may also support the host immune response against infection [117]. High-fiber diets may promote Firmicutes colonization in the GI tract, as this phylum is involved in converting complex fiber-based polysaccharides into their respective metabolites, such as SCFAs, supporting surrounding bacterial populations [118,119].

Fiber fermentation in the gut is a key indicator of gut health [120]. Still, a high level of fiber is unsuitable for children or neonate animals as a stimulator of beneficial bacteria to cope with cryptosporidiosis; however, probiotics could be an alternative in these cases [121]. The therapeutic effect of probiotics such as *Enterococcus faecalis* CECT7121 on *C. parvum* infection could be attributed to competition for binding sites on the gut epithelium, the acidification of the medium induced by lactic acid bacteria [122], an increase in the number of IgA-producing cells, or increased production of IgM [123]. Similarly, *Cryptosporidium* spp. infection in neonatal rats showed a trend where using probiotics during the infection led to more clearance of cryptosporidiosis; however, daily administration of *Lactobacillus casei*-containing mixtures in the neonatal rats model did not remove the protozoan [124]. Further research is needed to check the anti-*Cryptosporidium* effects of different probiotics.

Current studies suggest that dietary changes or probiotics may reduce cryptosporidiosis severity; however, they haven’t established guidelines for clinical use or assessed their potential as a treatment for the infection itself. Previous studies have primarily administered probiotics to the hosts before infection to reduce the severity of cryptosporidiosis. Further research is needed to determine the optimal timing for probiotic use, whether pre-infection to reduce the severity, post infection, or as a preventive measure against chronic gut diseases by rebalancing bacterial diversity [54].

### 4.1. Interrelations Between Crypsyposridiosis, Gut Microbiota-Derived Metabolites, and Immune Responses

The age of the host is also an important factor in the animal model, and should be considered when discussing gut microbiota variations. VanDussen et al. (2020) indicated that in the murine model, an almost fully established microbiome is formed within three weeks after birth [46]. This is significant, as the microbial alterations during cryptosporidiosis play an important role in gut metabolism. Numerous processes are involved in the *Cryptosporidium*–microbiome interactions [46].

Recent studies with mice by Charania et al. (2020) also demonstrated that in mice without antibiotics treatment, *Cryptosporidium* spp. infection increased *Lactobacillus* communities, whereas antibiotic pre-treatment with cloxacillin led to a decrease [125]. *Lactobacillus* spp. are known to act as beneficial bacteria and protect against infections, and have been reported to have a direct effect on *C. parvum* oocyst viability in vitro by producing antimicrobial substances [126]. Notably, the mice in this study exhibited no clinical signs of cryptosporidiosis, suggesting that they may have been asymptomatic carriers of the parasite. In contrast, studies of goats exhibited mild to severe cryptosporidiosis symptoms, e.g., hypothermia, loose feces, inhibited growth, and death, and have reported a depletion of bacterial species involved in SCFA production, disrupting SCFAs’ biosynthetic processes [127]. Short-chain fatty acids (propionate, butyrate, and acetate) are the major byproduct of gut bacteria. They break carbohydrates that are indigestible and are recognized as vital energy materials, have anti-cancer and anti-inflammatory characteristics, can lower cholesterol and fat storage, regulate the pH of the intestine, and also avoid predatory harmful germs from entering and sticking to gut surfaces [128].

Reduced levels of SCFAs in infected hosts have been linked to an altered host microbiome [100,129]. Increased concentrations of SCFAs are crucial metabolites produced by gut bacteria through the fermentation of dietary fibers, which have shown direct inhibitory effects on *C. parvum.* For instance, in vitro studies demonstrated that butyrate, acetate, and propionate significantly inhibited the growth of *C. parvum* when administered to infected human intestinal cells [130]. Notably, butyrate not only reduced parasite proliferation, but also increased apoptosis in infected enteric cells, suggesting an underlying mechanism for how increased SCFA concentration inhibits *Cryptosporidium* spp. infections. Additionally, SCFAs play an important role in maintaining gut epithelial integrity, as the production of mucus and strengthening of TJs between epithelial cells were found to be positively influenced by increased SCFA concentration, hence preventing the invasion of pathogens and maintaining intestinal barrier integrity [87,131].

Also, the production of SCFAs was found to be linked to increased synthesis of anti-inflammatory cytokines and modulation of immune reactions, thereby supporting local and systemic immunity against infections [130]. Short-chain fatty acids affect the activity of different immune cells, e.g., regulatory T cells (Tregs), type 3 innate lymphoid cells (ILC3s), CD4+ T cells, and Th1e ectorcells that generate IFN-γ [132]. IFN-γ is an important immune factor in controlling cryptosporidiosis [133]. It has been proved that in IFN-knockout mice or mice treated with anti-IFN-γ-antibodies, the vulnerability to *C. parvum* invasion was increased, and, in such mice, the parasitic shed also increased [134]. It has also been observed that mice that were lacking in B and T cells showed reduced *C. parvum* infection compared to IFN- γ-knockout mice, demonstrating that a part of IFNs during *C. parvum* infection is developed from non-T and B cells [135]. Experiments with piglets and in vitro cell lines have shown that intestinal epithelial cells secrete a large number of type-III IFNs, independent of specialized immune cells. Initially, the literature indicated that type-III IFNs had been linked with epithelial defense against viruses, but later it was proven that IFNs are involved in defense mechanisms against non-viral infection, also via TLRs [136,137]. Priming epithelial cells of the gut with recombinant IFN-λ3 results in less barrier disruption and enhanced cellular resistance against *C. parvum* infection [136].

Butyrate regulates gut bacterial ecology, while propionate upregulates NF-κB and IL-18 [138]. Butyrate is also a histone deacetylase inhibitor and is involved in cell cycle and cell proliferation [139]. Interestingly, butyrate, propionate, and acetate are involved in the upregulation of IL-18 [140]. The immunomodulatory effects of SCFAs include upregulating the production of anti-inflammatory mediators while suppressing pro-inflammatory cytokines, thus helping to balance immune responses during cryptosporidiosis [141]. For instance, CD4+ T cells, which are actively influenced by SCFAs, are critical players in eliciting appropriate immunological responses to cryptosporidiosis [142]. CD4+ T immunological cells play an important role in clearing *Cryptosporidium* spp. throughout the acute phase of an infection (which includes innate immunity) [143]. The first group of CD4+ T helper cells comprises TH17 cells that develop on antigen presentation cells (APCs) that are stimulated by pathogens, and are hence crucial during the initial phases of infection [144]. TH17 cells develop from immature CD4+ T cells in the presence of transforming growth factor beta (TGF-β) and IL-6, and IL-23 promotes TH17 cells to produce IL-17 rather than IL-4 or IFN-γ [144]. IL-17, through its involvement in chemokine and cytokine production, positively influences chemoattraction (for neutrophils) at the infection site, and IL-17 plays a role in immune reaction by facilitating innate immune response against infectious agents [144]. Additionally, TH17 cytokines IL-6, IL-17, IL-23, TNF-α, and TGF-β have been detected in increased concentrations in the GALT (gut associated lymphoid tissues) and spleen of *C. parvum*-infected immunodeficient BALB/c mice [145].

Lactate metabolizing bacteria actively produce D-amino acids [146], enhancing the microbial response to mitigate mucosal and epithelial damage during cryptosporidiosis [147]. D-amino acids mediate microbiome host cross-talk, [96,97] with producers like D-amino acid-producing species, such as Lachnospiraceae, *Marvinbryantia* spp., *Lactobacillus* spp., and *Lachnoclostridium* spp. in the mouse colon [148,149]. However, the results indicated that during cryptosporidiosis, the microbiome produces high levels of D-amino acids in the small intestine [100]. Infected mice showed increased levels of *Lachnoclostridium* spp., Lachnospiraceae, and *Lactobacillus* spp., particularly in duodenum and jejunum, suggesting their role in the production of D-amino acids, consistent with findings by Sasabe et al. (2016) [149].

It was found that during a given parasitic infection, the citrate cycle in the intestine was more active [100]. *Cryptosporidium parvum* lacks the citrate cycle and relies on salvaging metabolites from the host gut [150]. Research results have demonstrated that microbial carboxylase transporter proteins are implicated in promoting colonization and pathogenicity in bacteria like *Salmonella enterica* and *Haemophilus influenzae*. This process utilizes energy sources like glutamate, leading to elevated levels of dicarboxylic acids such as hexanoate and acetate [151]. In this context, *Cryptosporidium* spp. excystation in the duodenum has been linked to an increased expression of proteins involved in glycolysis, glutaminolysis, and the citrate cycle in the small intestine [152]. In the infected caecum, glutamate metabolism was elevated, likely due to glucose depletion, promoting *Cryptosporidium* and host defense cells to use glutamate as a primary carbon source. [153,154]. During cryptosporidiosis, glutamate utilization to generate α-ketoglutarate, facilitated by enzymes like glutamate kinase, glutamate-5-semialdehyde dehydrogenase, and glutamine synthetase, was observed [153]. Upregulated host and yeast transketolases, along with yeast polyubiquitin proteins, were detected, suggesting their role in catalyzing ubiquinone biosynthesis in the jejunum–ileum tract [100].

Ubiquinone biosynthesis begins with erythrose 4-phosphate (E4P) metabolism, catalyzed by glucose-6-phosphate dehydrogenase (G6PDH), during infection by trypanosomatid, *C. parvum* [155], and *Plasmodium* spp. [156]. Enzyme activities in the yeasts and host indicate a host–parasite–microbiome interaction in the small intestine. This interaction may compensate for *Cryptosporidium* spp.’s deficient metabolic machinery in ubiquinone (coenzyme Q) synthesis, which is essential for the electron transport chain. Similar associations supporting *Cryptosporidium* spp.’s multiplication have been reported in aquatic environments [157] and neonatal mice with gut dysbiosis [46]. Elevated yeast ubiquitin-related activity, mediated by ubiquitin-conjugating enzymes, was detected throughout the small intestine, particularly in the ileum. These proteins are essential for ubiquinone’s metabolism, synthesis, and transfer [153,156]. It is believed that *Cryptosporidium* spp. rely upon the host ubiquinone system for salvage [153].

Also, the host actin plays a crucial role in the invasion and replication of apicomplexan parasites like *Cryptosporidium* spp. [158]. Previous microscopy and cell culture studies have demonstrated that *Cryptosporidium* spp. induces the assembly and polymerization of the host actin into plaque structures, facilitating sporozoite invasion [159]. Notably, the upregulation of the actin in response to parasite infection has not been previously reported. In the context of cryptosporidiosis, the increased expression of the actin in the mouse gut is a novel finding that warrants further investigation through targeted proteomic studies.

### 4.2. Mechanisms Linking Microbiota to Gut Barrier Integrity in Cryptosporidiosis

The established results suggest that certain microbial populations play a vital role in maintaining TJs’ integrity. For example, studies have demonstrated that germ-free mice showed lower expression of occludin and claudin-4, increasing their vulnerability to epithelial injury [160]. Conversely, a diverse microbiome enhances mucin and antimicrobial peptide secretion, strengthening the mucus layer and supporting TJs’ stability [161]. The gut microbiota shapes mucosal immunity through interactions with epithelial cells, which is crucial for balancing immune tolerance and effector functions. For instance, certain bacterial populations boost the production of immunoglobulins (e.g., IgA), aiding in pathogen defense by blocking their adhesion to epithelial cells [162]. During cryptosporidiosis, a disrupted gut microbiome composition leads to dysbiosis, with reduced diversity and a shift in bacterial populations, exacerbating inflammation and weakening mucosal immunity [54,163]. During cryptosporidiosis, altered microbiota increased intestinal permeability and weakened immune responses, emphasizing the connection between microbial balance, health, and host defense [54,100]. Cryptosporidiosis disrupts the gut microbiome, increasing pathogenic bacteria while reducing beneficial species. This dysbiosis drives persistent inflammation and hampers recovery [100,163]. Additionally, altered SCFA production during cryptosporidiosis impacts epithelial integrity and immune response modulation, further exacerbating disease severity [100].

## 5. Future Research Directions

Future researchers should use high-throughput sequencing techniques to characterize the gut microbiota composition before and after *Cryptosporidium* spp. infection. This can lead to the identification of specific microbial communities and their relationship to health, disease severity, and outcomes. Current research indicates that increased diversity in the gut microbiome can potentially diminish clinical signs and oocyst shedding during infection [164]. Additionally, research should also explore the specific processes and micro-details through which *Cryptosporidium* spp. induced impairments in TJs’ integrity. Uncovering how the parasite interacts with host enteric epithelial cells and modulates inflammatory cascades can lead to the discovery of therapeutic agents. Previous studies have proven that *Cryptosporidium* spp. shift gut microbiota, worsening barrier dysfunctions [163].

Research has also demonstrated that infection reduces SCFA levels, contributing to dysbiosis and dysfunction of the intestinal barrier [100]. Future research could explore the effect of dietary interventions to boost SCFA production and the restoration of gut health after infection. Also, the potential of fecal microbiota transplant (FMT) to manage chronic cryptosporidiosis warrants evaluation. Clinical trials are needed to evaluate interventions for restoring microbial balance and improving outcomes during infection [100]. Chronic cryptosporidiosis in individuals with compromised immunity underscores the need for treatments targeting gut microbiome modulations [165]. Dietary modifications to enhance gut barrier functions during *Cryptosporidium* spp. infection offer a promising research avenue. Particular attention should be given to high-fiber diets, which have been demonstrated to improve microbial diversity and mucosal immunity [115]. Investigating how dietary components affect microbiota composition and host responses during infection could provide valuable future research directions.

Longitudinal studies in vulnerable communities like immunocompromised patients and children are crucial for understanding the long-term effects of cryptosporidiosis on microbiota variations and intestinal barrier integrity. This will aid in developing targeted interventions for chronic and recurrent infections [166].

## 6. Conclusions

*Cryptosporidium* spp. are a significant threat to both public health and the livestock sector because of severe damage to intestinal barrier integrity and reduced gut commensal microbes. This review has demonstrated the multi-dimensional impairments from cryptosporidiosis, e.g., the downregulation of TJPs, initiation of autophagy, and inflammatory mechanisms that negatively affect gut barrier functions. Also, the infection leads to variations in the gut microbiome and dysbiosis, negatively influencing the SCFA-producing bacteria (e.g., Firmicutes) and increasing the relative abundance of pathogenic bacteria, e.g., Proteobacteria. Innovative therapeutic options, e.g., probiotics supplementation, fecal microbiota transplantation, and dietary interventions, can prove promising in alleviating the hazardous effects of cryptosporidiosis. However, for their practical applications, further evidence-based protocols are needed. Future researchers should explore the intricate interrelations between the parasite and host microbiome through metabolomics and metagenomics analysis, using high-throughput sequencing to explore novel treatment options against cryptosporidiosis. Overall, addressing cryptosporidiosis from the perspective of the One Health approach, with special emphasis on interactions between animals, humans, and environmental health, is crucial to reduce the global burden of this parasitic disease.

## Figures and Tables

**Table 1:** Interrelations between cryptosporidiosis, gut barrier integrity with regard to TJPs, and other pathological alterations during *Cryptosporidium* spp. infection.

Animal Model	*Cryptosporidium* spp.	Effect of *Cryptosporidium* spp. Infection on TJs’ Integrity	Other Pathophysiological Alterations	References
Neonatal mice	*C. parvum*	Cell junction and adherens junction genes were downregulated at peak infection time, e.g., at 11 and 30 dpi, there was a decreased expression of E-cadherin	The V:C * was significantly reduced. An exaggerated inflammatory response and increased intestinal permeability resulted in diarrhea. Cell adhesion molecules were found to be involved in modulating apoptosis	[51]
Calves	*C. parvum*	Hindered cell junctions, such as AJs *, TJs *, GJs *, hemidesmosomes, and desmosomes	Increased inflammation, apoptosis, villus atrophy, and lowered V:C resulted in an increased intestinal permeability leading to diarrhea	[85]
Human small intestinal epithelial cells	*C. hominis*	Disruption of TJs and AJs, altered barrier function, or distribution of junctional proteins were detected	Many inflammatory genes were upregulated and intrinsic and extrinsic apoptotic pathways were activated, resulting in a disrupted intestinal barrier and diarrhea	[86]
Mice	*C. muris*	Infection altered epithelial AJs, which were mediated by E-cadherin	Reduced V:C and increased mucosal inflammation, mucosal thickness and villi diameter, alterations in gut bacteria composition, and production of metabolites (SCFAs) were observed	[87]
Mice	*C. parvum*	Infection downregulated TJ proteins occludin and claudin, which altered TJs between intestinal epithelial cells	Infection upregulated the expression of inflammatory genes, e.g., Nos2, Mip2, and Icam1, hence boosting immune responses inducing villus atrophy and inhibiting intestinal enteroids via increased apoptosis. The release of stress signals that can inhibit intestinal stem cell function in the crypt was also detected	[88]
PEC (Human Primary Intestinal Epithelial Cells) culture media	*C. parvum*	Affected the intestinal barrier by disrupting the assembly of TJs	Modulated inflammatory response, damaged microvilli, and increased epithelial permeability	[89]
Human Intestinal Enteroids (HIE)	*C. parvum*	Modified protein or RNA expression related to TJs and AJs	Host cell nuclei were constricted or collapsed, indicating apoptosis. Polarized apical brush border microvilli displayed disrupted TJs with an increased basolateral expression of NA^+^/K^+^ ATPase	[74]
Mice	*C*. *parvum*	Intestinal epithelial barrier dysfunctioning due to the disruption of epithelial junctional complexes was found	Increased mucosal infiltration with neutrophils and a marker of inflammation in the ileum, epithelial cell apoptosis, shortening of ileal and jejunal villi, increased permeability, and diarrhea were observed	[50]
Caco-2 cell monolayers as in vitro model of IECs *	*C*. *parvum*	Disruption of intestinal epithelial barrier function as a result of significant downregulation of critical epithelial TJ and AJ proteins was observed	Polymorphisms in autophagy genes, impaired immune responses, and epithelial cell function led to enteritis. Autophagy in absorptive cells, e.g., Paneth and goblet cells, was observed	[90]
Mice	*C*. *parvum*	Downregulated the mRNA expression levels of ZO-1 *, claudin 3, and occludin and compromised the integrity of the intestinal barrier by downregulating TJs	Pathological damage led to necrotic enteric epithelial cells, shorter villi, a lower V:C, and an increased villi diameter. Suppression of C3aR * worsened the damage, further reducing intestinal permeability	[91]
Human ileal adenoma cell model HCT-8	*C*. *parvum*	Absence of adherence to neighboring cells and disrupted expression of proteins such as integrins and cadherins were detected	Modulated inflammatory response, cell proliferation, differentiation, apoptosis, altered gene expression, and shortened villi with an increased mitochondrial membrane permeability, causing potential anomalies and releasing apoptogenic substances like cytochrome C, were detected	[92]
Human and bovine epithelial cells	*C. andersoni*	Disruptions of ZO-1 that serves as a connection between TJ occludin and cytoskeletal F-actin were detected	Parasite invasion triggered enteritis and apoptosis. These disturbances lead to cytotoxic effects on enterocytes, resulting in increased intestinal permeability and loss of the barrier function	[93]
Neonatal mice and dogs	*C. canis*	The integrity of the epithelial cells of BF * was somewhat damaged	Inflamed and disordered epithelial surfaces led to cell death. Additionally, the compromised microvillus border resulted in the loss of cilia and atrophied mucosa in the duodenum and jejunum, and hence, increased intestinal permeability	[94]
Mice	*C*. *parvum*	Disrupted the AJ complex between intestinal epithelial cells	Ly6C+ * inflammatory monocytes induced the production of TNF-α and IL-1β. The infection triggered apoptosis, leading to epithelial cell loss, villi blunting, and shortening	[95]
Cattle	*C. parvum*	Disrupted epithelial microvilli and TJs between epithelial cells	Dysregulated chemokine and cytokine production exacerbated inflammation. Infection triggered apoptosis and increased mast cells in jejunal villi, leading to villi damage and increased enteric epithelial permeability	[96]
Neonatal calves	*C. parvum*	Weakened TJs between epithelial cells	Enteritis, especially with concurrent bacterial or viral infections, damaged villi and microvilli led to leaky gut syndrome	[97]
Mice	*C. parvum*	Negatively affected epithelial cell junctions disrupted ZO-1 in Caco-2 cells	T cell-mediated inflammation caused epithelial cell damage through parasite invasion, proliferation, and extrusion, resulting in villus atrophy, crypt hyperplasia, epithelial cell loss, and villi blunting and shortening, with increased inflammatory cell infiltration in the crypts and weakened local mucosal immunity	[98]
Piglets	*C. parvum*	Infected enterocytes retained their capacity to regulate TJs	Infected tissue mucosa showed increased inflammatory cells, particularly in the lamina propria, leading to epithelial cell damage through apoptosis and resulting in shortened villi and altered epithelial macromolecular permeability	[99]

* in the table text indicates that the full form of the abbreviation used or an explanation regarding the given abbreviation is mentioned in the footnote. V:C: villi-to-crypt ratio; TJs: tight junctions; AJs: adherens junctions; GJ: gap junctions; IECs: intestinal epithelial cells; ZO-1: zonula occludin; C3aR: C3a (complement component 3a) receptor; BF: bursas of Fabricius; Ly6C+: Lymphocyte antigen 6 complex locus C positive.

## Data Availability

No new data were created or analyzed in this study because it is a comprehensive review.
